# The spatial and dynamic impact of air pollution on public health: Evidence from China 2000–2021

**DOI:** 10.1371/journal.pone.0327319

**Published:** 2025-07-07

**Authors:** YuMing Qin, XueQin Lin, Ye Duan

**Affiliations:** 1 College of Resources Environment and Tourism, Capital Normal University, Beijing, China; 2 School of Geography, Liaoning Normal University, Dalian, Liaoning, China; Lanzhou Jiaotong University, CHINA

## Abstract

China’s rapid economic growth and improving quality of life have led to severe air pollution, primarily due to the country’s development model. This pollution not only raises public health risks but also shortens life expectancy, drawing significant attention from both the public and the government. This study focuses on 31 provincial-level regions within China, utilizing data collected annually from 2000 to 2021. It begins by examining the spatial relationships between air pollution and public health, then delves into how air pollution and various influencing factors affect public health outcomes. Lastly, the research investigates how these effects vary across different regional contexts. The findings show a clear connection between the medical visits for diagnosis and treatment and the levels of air pollution across different provinces. The spatial econometric model reveals that PM_2.5_ levels, industrial SO_2_ emissions, and smoke and dust emissions from industries all significantly increase medical visits for diagnosis and treatment. A 1% rise in PM_2.5_, SO_2_, or industrial smoke and dust emissions leads to increases of 0.2884%, 0.0563%, and 0.1365%, respectively, in medical visits. This suggests that air pollution contributes to a decline in public health. The impact of air pollution on public health shows considerable variation across different regions, including the eastern, central, and western parts of the country. The results of this study offer fresh insights into how air pollution affects public health, providing important guidance for policies aimed at improving air quality and protecting the health of citizens.

## 1. Introduction

Human health forms the foundation of both individual well-being and societal progress [1]. As the global economy and society expand rapidly, the environment has suffered significant damage, with pollution becoming a serious threat to human health. According to Juginovic et al., air pollution is identified as one of the top global health risks, ranking fourth in terms of its impact on public well-being [2], and access to clean air is considered essential for maintaining good health and quality of life. By 2019, almost the entire global population, or 99%, lived in areas where air quality surpassed the regulatory limits [3]. Research has repeatedly demonstrated that air pollution poses a significant threat to public health, with its effects leading to the loss of more than 100 billion disability-adjusted life years (DALYs) globally every year. Disability-adjusted life years refer to the entire loss of healthy life years from disease onset to death, including those caused by premature death and those caused by disease [4]. In recent decades, there has been growing global concern over the potential health effects of environmental pollution. Nearly 25% of human infections are brought on by prolonged exposure to environmental pollutants, and the majority of these illnesses are latent and challenging to diagnose, according to the World Health Organization [5]. In other words, striking a balance between environmental preservation, public health advancement, and economic expansion is a clear global policy problem. Therefore, a key discussion centers around finding ways to achieve a balanced and sustainable relationship between the natural environment and human progress.

Scholars are increasingly focusing on how air pollution affects public health, recognizing it as a critical area of study [6]. Various fields, including medicine, environmental epidemiology, and geography, have conducted detailed studies on how air pollution affects public health. These disciplines offer different insights based on their unique areas of focus [7]. Research on how air pollution affects public health primarily examines two key aspects: Firstly, the internal relationship between individual spatiotemporal behavior and air pollution was analyzed. Secondly, the mechanism of individual spatiotemporal behavior trajectory on air pollution exposure and its impact on health was explored at the micro level [8]. Huang et al. monitored the air pollution index of different modes of transportation at different times using real-time PM_2.5_ monitoring devices [9]. The research results showed that compared with residents who take buses and ride bicycles, residents who commute by taxi have significantly lower levels of air pollution exposure. On the other hand, at the macro-regional scale, recent studies have increasingly focused on how air pollution correlates with various health indicators [10]. This type of research mainly relies on theoretical frameworks and analytical methods from multiple disciplines such as environmental medicine, geography, and epidemiology. From a macro perspective, it deeply analyzes the spatiotemporal evolution trajectory of natural factors and air pollution sources, and further explores the potential impact laws of these evolution processes on human health, providing a basis for formulating targeted environmental protection and health promotion strategies at the macro level [11–14]. Sun Meng et al. introduced air pollution factors and conducted research using smoke and dust emissions as proxy variables. The findings of the study suggest that air pollution from particulate matter, driven by smoke and dust emissions, has negatively impacted the health of residents in China. However, this negative effect has notably diminished when factors such as economic development and green spaces are taken into account [15].

Although previous studies have extensively explored the effects of air pollution on public health across different regions, few have adequately considered the broader regional impacts and spillover effects [6]. It is important to remember that air pollution affects the surrounding areas indirectly in addition to directly [16]. The impact of air pollution on public health is bound to spread beyond the local area, affecting the health conditions of nearby regions [10]. It is insufficient to rely only on neighborhood initiatives to enhance air quality, the interaction between regions and consideration of the spatial dependence of surrounding areas is crucial [17]. It is necessary to have a multidimensional understanding of the differences in the regional economy, society, environment, and health, as well as the impact of air pollution [18]. The impact of air pollution, along with other factors, on public health differs significantly across regions and locations [19]. From the perspective of new economic geography, examining the geographical factors and considering how air pollution spillovers impact public health is increasingly important and valuable [20]. Therefore, this study adopts a spatial geography framework to examine how air pollution evolves over time and across locations, assess its effects on public health, and supplement the research gap in the field of spatial geography.

China presents a practical framework for examining how air pollution affects public health. The nation has undergone swift urbanization and significant economic growth [21]. Over the past four decades of economic reforms, China has consistently achieved a remarkable annual growth rate, surpassing 9% [22]. While China’s rapid economic growth has led to impressive progress, it has also uncovered numerous environmental issues, with air pollution becoming a major concern for public health [23]. At the 19th National Congress of the Communist Party of China, General Secretary Xi Jinping highlighted the vital role of the Healthy China initiative. He underscored that protecting the health of the populace has now achieved a status of national priority, thus laying the groundwork and providing essential support for designing the 14th Five-Year Plan and the 2035 Long-Range Objectives Outline [24]. This article focuses on 31 provincial-level regions in China, examining annual data spanning from 2000 to 2021. This study examines the connections between air pollution and public health, evaluates the impact of air pollution and other influencing factors on health outcomes, and explores regional differences. The aim is to establish a scientific foundation for enhancing air quality and protecting public health.

This study makes several important contributions: (1) It adopts a geographical lens to examine how air pollution affects public health, taking into account both the spatial spread of pollutants and the regional interconnections related to health outcomes. It should be noted that previous studies often overlooked the potential correlation effects between factors, resulting in biased evaluation results. (2) This article offers a thorough examination of how air pollution affects public health, taking a provincial approach while also considering its geographical implications across eastern, central, and western China. It serves as a crucial resource for relevant authorities, helping them craft tailored strategies that reflect the distinct characteristics of each region, and also has some innovative aspects. As a result, the findings of this research aim to offer a scientific foundation or guide for fostering regional collaboration on air pollution mitigation and the development of healthier living environments across the nation. (3) Adopting an interdisciplinary approach, this research utilizes a socio-economic framework to tackle issues related to public health and environmental pollution, aiming to more accurately manage overlooked confounding variables.

This paper is structured as follows: In Section 2, the research methodology and data sources are outlined. Section 3 follows with an analysis of the findings, accompanied by a discussion. Lastly, Section 4 concludes with a summary and offers policy suggestions.

## 2. Materials and methods

### 2.1 Model specification

This section presents Moran’s *I* index, which we use to examine the spatial connections between air pollution and public health outcomes in China [25]. Building upon this, we employ the spatial Durbin model to investigate the interactions between these two factors over time.

#### 2.1.1 Spatial correlation test.

Before formally implementing quantitative regression analysis, spatial autocorrelation testing of data is crucial. Currently, in spatial econometric analysis, Moran’s *I* for testing is commonly used autocorrelation test method. Moran’s *I* is classified into two types according to the spatial scope of analysis: the global Moran’s *I*, which reflects overall spatial patterns, and the local Moran’s *I*, which focuses on specific regions. To present the global situation more intuitively, it is usually necessary to draw a local scatter plot.

The assessment of spatial relationships for an individual variable is commonly carried out through the application of global and local Moran indices. Other statistical measures frequently employed in spatial analysis include indices such as Geary’s C and Moran’s *I* [26], among others. In this study, the focus is primarily on the use of Moran’s *I* to quantify the degree of spatial association [27,28]. The calculation can be determined using the following equation:


$$I=\frac{\sum_{i=1}^{n}\;\;\sum_{j=1}^{n}\;\;W_{ij}\left(x_{i}-\bar{x}\mathrm{\backslash rightleft}(x_{j}-\bar{x}\right)}{S^{2}\sum_{i=1}^{n}\;\;\sum_{j=1}^{n}\;\;W_{ij}}$$ (1)

Among them, *I* denotes the global Moran’s *I* statistic, *n* refers to the total count of sample areas, *x*_*i*_, and *x*_*j*_ are the values observed in regions *i* and *j*, respectively, the symbol $\bar{x}$represents the mean of the observed data across all regions, and *S*^*2*^ is the variance of observed variables in all regions [29,30]. The range of *I* value is between −1 and 1. Within a given area, higher values tend to cluster together, as do lower values, particularly when the index exceeds 0. This suggests a positive spatial correlation between the values observed across different regions. The data collected for each region have a geographic negative correlation when the index is less than zero, meaning that high and low values are adjacent within the region; if the index is zero, the recorded values have no spatial correlation [31].

The matrix representing spatial adjacency, denoted as *W*, is used in this study. In this empirical investigation, the matrix helps identify whether areas within the research region are spatially neighboring, by assigning binary values of 0 or 1. Specifically, when two regions share a common boundary, the corresponding values in the matrix are assigned a value of 1, reflecting their adjacency; If there is no common boundary, the values of the corresponding elements are set to 0, indicating that they are not adjacent [32]. In addition, in the process of conducting spatial econometric model regression, to ensure the accuracy and consistency of the analysis, the spatial weight matrix was standardized [33]. The specific formula is shown below.


$$\begin{array}{c} W_{ij}=\left\{\;\;\;\begin{array}{c} 1Regioni\text{and\textbackslash{} region}j\text{are\textbackslash{} adjacent}\\ 0Regioni\text{and\textbackslash{} region}j\text{are\textbackslash{} not\textbackslash{} adjacent} \end{array}\;\;\;\right\} \end{array}$$ (2)

The Local Moran’s *I* statistic is a useful tool for assessing the presence of spatial dependence in a given dataset. Its formula for computation is as follows:


$$I_{i}=\frac{\left(x_{i}-\bar{x}\right)}{S^{2}}\sum_{j=1}^{n}\;W_{ij}(x_{j}-\bar{x}\backslash$$ (3)

In the formula, the value of Moran’s *I* typically ranges between −1 and 1. Usually, Moran’s *I* shows positive spatial autocorrelation, meaning that nearby values are more similar to each other. This index quantifies the relationship between observed values and their spatially lagged counterparts. Additionally, Moran’s *I* can be illustrated using a scatter plot, where the regression line’s slope reflects the magnitude of this spatial autocorrelation.

Expanding on this idea, when performing a bivariate spatial correlation analysis, the bivariate global Moran’s index offers an enhanced approach compared to the standard Moran’s index. It measures the relationship between two different variables—one located in a specific spatial unit and the other in a neighboring unit—allowing for a more nuanced understanding of spatial associations [34,35]. To evaluate how air pollution in one province affects the health of neighboring areas, the bivariate global Moran’s *I* statistic is employed [36]. The following equation represents the calculation method:


$$I_{kl}=\frac{\sum_{i=1}^{n}\;\;\sum_{j=1}^{n}\;\;W_{ij}\left(x_{k}^{i}-\overset{―}{x_{l}}\mathrm{\backslash rightleft}(x_{k}^{j}-\overset{―}{x_{l}}\right)}{\left[S^{2}\sum_{i=1}^{n}\;\;\sum_{j=1}^{n}\;\;W_{ij}\right]}$$ (4)

In this equation, *I*_*kl*_ denotes the bivariate global Moran’s index, the term $x_{k}^{i}$ corresponds to the measurement of the *k* variables at spatial location *i*, $x_{k}^{j}$ indicates the corresponding measurement at spatial location *j*.

#### 2.1.2 Spatial econometric model.

This research proposes a spatial econometric model to examine how air pollution impacts public health outcomes. The first step before starting spatial econometric analysis work is to conduct a detailed validation process on the adopted spatial econometric model. Anselin’s study categorizes spatial measurement models into three types [37]: the spatial error model (SEM), the spatial lag model (SAR), and the spatial Durbin model (SDM). When evaluating different models, it’s important to account for whether the effects are spatially fixed, temporally fixed, or both fixed over time, while also considering the interchangeability of fixed and random effects. These issues need to be determined through testing. Firstly, the LM test method is applied and combined with SEM and SAR models for selection. If the LM error test passes but the LM tag test fails, the SEM model is a more suitable choice [38]. On the contrary, if the LM tag test passes but the LM error test fails, the SAR model should be used [39]. If the results of both tests are not significant, further judgment needs to be made through R-LM tag and R-LM err tests. If the R-LM-err test passes but the R-LM-lag test fails, the SEM model is more appropriate [40]. On the contrary, the SAR model is a better choice. If all Lagrange significance tests fail, the spatial Durbin model should be the preferred solution for econometric analysis.

To determine whether to use the fixed effects or random effects models, the Hausman test is applied. When using fixed effects models, LR tests are used to confirm if the model should incorporate spatial or time fixed effects. Table 1 displays the appropriate test findings. Following testing, we decided to use a double fixed space Durbin model in this study to examine how air pollution affects public health. The model in question is as follows:

**Table 1:** LM, LR, and Hausman test results of spatial econometric models.

	Testing method	Statistical value	P value
Selection of SEM and SAR models	LM-lag	13.819	0.000
	R-LM-lag	23.486	0.000
	LM-error	61.877	0.000
	R-LM-err	58.544	0.000
SDM model degradation test	LR(SDM&SLM)	127.52	0.000
	LR(SDM&SEM)	129.95	0.000
SDM fixed effects test	Spatial-LR	136.75	0.000
	Time-LR	1428.21	0.000
SDM random effects test	Hausman	110.11	0.000


$$Y_{it}=a_{it}+\rho W_{ij}Y_{it}+\beta X_{it}+\delta W_{ij}X_{it}+u_{i}+\delta_{i}+\varepsilon_{it}$$ (5)

In the formula, the indices *i* and *j* refer to specific provinces, while *t* stands for the year. The parameter *ρ* denotes the spatial autoregressive coefficient [41], which measures the degree to which the outcome variable *Y* is influenced by the values of adjacent spatial units. *X* denotes the set of explanatory variables, while *W* denotes a spatial weight matrix derived using the Queen’s contiguity criterion; the variable *WY* represents the spatial lag effect of the outcome variable, and *β* signifies the impact of the explanatory variables on the outcome variable; the parameter *δ* is the coefficient associated with the spatial lag term of the independent variable, denoted as *WX*; the variables *u*_*i*_ and *δ*_*i*_ represent the fixed effects at the individual level and the time level, respectively. Finally, *ε* represents the stochastic error term, which is assumed to be normally distributed.

### 2.2 Variable selection and data sources

#### 2.2.1 Variable selection.

The dependent variable is public health. Its connotation is extremely rich, involving multiple complex endogenous mechanisms, making direct measurement of health quite difficult. Previous studies in the field of public health often rely on various metrics to assess the health status of populations. These measures include general mortality figures, mortality rates among young children, and life expectancy at birth, which together offer a broader understanding of public health trends [42]. The factors contributing to human death are often complex and diverse. The amount of dust and suspended solids in the air has greatly grown as air pollution has gotten worse [6]. This phenomenon not only affects patients with chronic cardiovascular and cerebrovascular diseases and potential respiratory system diseases, but also increases their risk of illness or hospitalization [43]. Meanwhile, as residents’ awareness of the harm of air pollution to their health increases, it will encourage them to visit hospitals more frequently for health checkups. To provide a more accurate representation of how air pollution affects public health, this study uses the number of medical visits as a key measure of residents’ health, considering both data availability and the multifaceted effects of pollution on health.

Air pollution has been chosen as the primary explanatory variable. Core explanatory variables that can accurately reflect the state of air pollution, such as concentrations of PM_2.5_, emissions of industrial sulfur dioxide (SO_2_), and releases of industrial smoke and dust, were chosen in light of the complexity of the various types of air pollutants and the availability of data across different provincial regions. An increase in the levels of PM_2.5_, along with higher emissions of sulfur dioxide (SO_2_) and industrial particulates, can greatly elevate the risk of developing respiratory and circulatory diseases [44].

The control variables encompass seven factors, including GDP per capita, the share of the secondary sector in the economy [45], and the percentage of urban areas covered by greenery, local financial medical and health expenditures, number of practicing doctors, local financial education expenditures, and proportion of high school graduates. ①Economics. Per capita GDP is chosen as a metric to assess the income levels of residents [46], aiming to explore how regional economic conditions influence public health outcomes. ②Society. During periods of rapid economic growth, the increasing share of the industrial sector often leads to environmental degradation, creating significant challenges and placing considerable strain on natural resources [47]. Furthermore, industrial activities that rely on fossil fuels are a major source of PM_2.5_ formation [48]. As the concentration of these fine particles rises, they pose a growing risk to the health of local populations [49]. This research evaluates the industry composition by examining the proportion of value added by the secondary sector relative to the overall regional GDP. Moreover, increasing green spaces in urban areas not only improves air quality but also significantly benefits the physical and mental health of residents. This study evaluates the residents’ quality of life across different regions by analyzing the extent of green space coverage within urbanized areas. ③Education. This article uses local fiscal education expenditure and the proportion of high school graduates to measure the education level in various regions. ④Medical care. Enhancing the quality of healthcare services available to residents can reduce the health hazards associated with air pollution, while simultaneously raising public health standards [50]. This research assesses regional healthcare capabilities by analyzing indicators such as the number of healthcare professionals per 10,000 people and local government spending on healthcare. Table 2 provides a summary of the key variables considered in this analysis.

**Table 2:** Definition and interpretation of variables.

Variable type	Variable	Variable code
Explained Variable	Number of diagnosis and treatment visits (10000 people)	NDT
Explanatory variable	PM_2.5_ concentration (micrograms/cubic meter)	PM_2.5_
	Industrial SO_2_ emissions (tons)	SO_2_
	Industrial smoke and dust emissions (ton)	SD
Control variable	Per capita Gross Domestic Product (yuan/person)	PGDP
	Proportion of secondary industry (%)	CYJG
	Green coverage rate in built-up areas (%)	GLP
	Local financial medical and health expenditures (in billions of yuan)	YL
	Number of practicing physicians (in 10000)	YS
	Local fiscal education expenditure (in billions of yuan)	JY
	Proportion of high school graduates (%)	GZ

#### 2.2.2 Data source.

**Fig 1:**
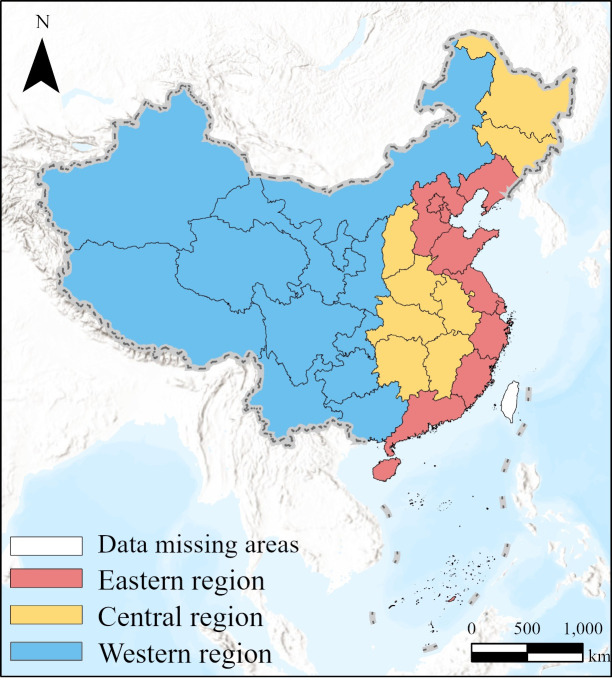
Study areas, along with the classification of China into eastern, central, and western regions.

Based on the availability of data, this study focuses on 31 provincial-level regions in China, as illustrated in Fig 1. The data utilized in this paper is primarily sourced from the China Health Statistics Yearbook (2001–2022), the China Statistical Yearbook (2001–2022), the China Environmental Statistics Yearbook (2001–2022), and the National Bureau of Statistics of China [51–54]. Since China’s PM_2.5_ concentration was only measured in 2013 [55], the PM_2.5_ concentration in each Chinese province from 2000 to 2012 was determined using the International Earth Science Information Network Center at Columbia University’s annual average global PM_2.5_ concentration from 2000 to 2012 [56]. Furthermore, to address the issue of heteroscedasticity that could distort the regression outcomes, a logarithmic transformation was applied to several variables, including the number of medical visits, industrial SO_2_ emissions, emissions of industrial smoke and dust, and per capita GDP. To account for price variations, the GDP deflator is applied to adjust per capita GDP to constant prices using the year 2000 as the base year [57]. Table 3 provides a summary of the descriptive statistics for the variables.

**Table 3:** Descriptive statistical results of variables.

Variable	Obs	Mean	Min	SD	Max
Hospital visits for diagnosis and treatment	682	8.52	5.3	0.98	10.6
PM_2.5_ concentration	682	43.41	5.5	18.26	112.7
Industrial SO_2_ emissions	682	12.43	6.6	1.56	14.35
Industrial smoke and dust emissions	682	12.19	6.84	1.3	14.19
per capita GDP	682	10.17	7.92	0.87	12.14
Proportion of secondary industry	682	41.97	15.97	8.36	61.96
Green coverage rate in built-up areas	682	36.42	6.6	6.15	49.3
Local financial medical and health expenditures	682	233.7	1.26	269.57	1857.1
Number of practicing physicians	682	8.6	0.39	6.04	34.28
Local fiscal education expenditure	682	520.64	6.49	531.54	3796.69
Proportion of high school graduates	682	0.52	0.1	0.17	0.89

## 3. Results and discussion

### 3.1 Spatial autocorrelation of number of diagnosis and treatment visits (NDT) and air pollution

#### 3.1.1 Global spatial autocorrelation test.

Table 4 displays the findings of the univariate global spatial correlation test between China’s public health and air pollution at the provincial level [10]. Industrial SO_2_ emissions, PM_2.5_ concentration in air pollution, the diagnosis and treatment visits, and industrial smoke and dust emissions all had Moran’s indices that were considerably higher than 0 and significant at the 10% level. This result strongly indicates a positive correlation between the spatial patterns of the variables. Over the period from 2000 to 2021, the global Moran’s index for medical visits fluctuated within a range of 0.101 to 0.234. Over the same timeframe, the global Moran’s index for PM_2.5_ concentrations showed a broader variation, ranging from 0.214 to 0.539. Regarding industrial emissions, the global Moran’s index for SO_2_ emissions varied between 0.112 and 0.206, while the index for smoke and dust emissions ranged from 0.128 to 0.343. The findings suggest a clear spatial relationship between PM_2.5_ levels, emissions of sulfur dioxide from industrial processes, industrial smoke and dust, and the number of medical visits. Upon examining the range of Moran’s index values, it becomes evident that the spatial autocorrelation of PM_2.5_ concentrations is particularly pronounced. Therefore, to fully understand the spatial impacts of air pollution on public health, it is crucial to consider the relationships between regions such as provinces and cities [21], rather than isolating each area as an independent unit.

**Table 4:** China’s univariate global Moran’s index of public health and air pollution.

Year	NDT	PM_2.5_	SO_2_	SD
2000	0.120^**^	0.214^**^	0.206^**^	0.165^**^
2001	0.124^**^	0.427^**^	0.196^**^	0.143^**^
2002	0.122^**^	0.386^***^	0.192^**^	0.148^**^
2003	0.101^**^	0.496^**^	0.145^**^	0.128^**^
2004	0.124^**^	0.395^**^	0.165^**^	0.135^**^
2005	0.126^**^	0.491^**^	0.193^**^	0.242^**^
2006	0.132^**^	0.439^**^	0.187^**^	0.275^**^
2007	0.119^**^	0.471^**^	0.198^**^	0.332^***^
2008	0.128^**^	0.455^**^	0.177^**^	0.343^**^
2009	0.169^**^	0.498^**^	0.157^**^	0.319^**^
2010	0.186^**^	0.388^**^	0.166^**^	0.252^**^
2011	0.176^**^	0.485^**^	0.203^**^	0.239^**^
2012	0.181^**^	0.424^**^	0.180^**^	0.193^**^
2013	0.185^**^	0.526^***^	0.164^**^	0.162^**^
2014	0.192^**^	0.504^**^	0.154^**^	0.270^**^
2015	0.198^***^	0.539^**^	0.144^***^	0.312^**^
2016	0.185^**^	0.519^**^	0.167^**^	0.326^**^
2017	0.198^**^	0.465^**^	0.132^**^	0.337^**^
2018	0.202^**^	0.398^**^	0.112^*^	0.339^**^
2019	0.195^**^	0.371^**^	0.119^*^	0.340^**^
2020	0.211^**^	0.305^**^	0.148^**^	0.339^**^
2021	0.234^**^	0.249^**^	0.155^**^	0.337^**^

Note:

* ,

** ,

*** respectively indicate passing the test at the 10%, 5%, and 1% significance levels.

#### 3.1.2 Local spatial autocorrelation test.

To gain deeper insights into the regional patterns of PM_2.5_ levels, industrial emissions of smoke and dust, and SO_2_ emissions, this paper presents local Moran’s *I* scatter plots for the four indicators: 2021 public health, PM_2.5_ levels, smoke and dust emissions from industry, and industrial SO_2_ emissions. These plots are used to examine the local spatial relationships between these variables. In the figure, the x-axis displays the values of the indicators, while the y-axis shows the corresponding spatial lag for each indicator.

**Fig 2:**
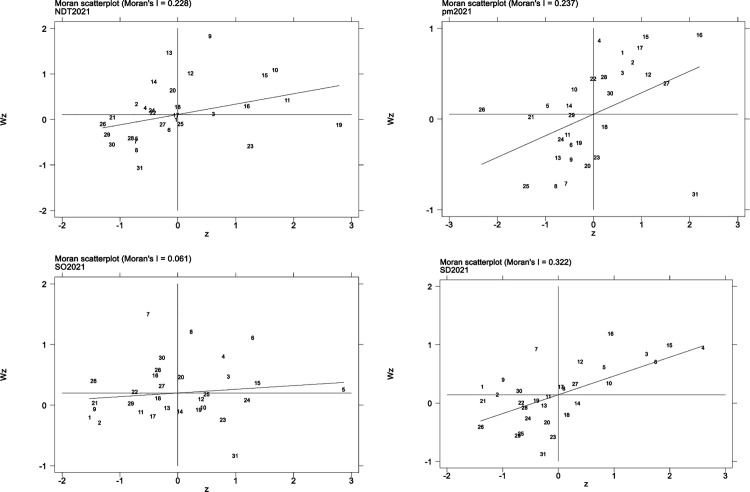
The local Moran’s index scatter plots for NDT, PM_2.5_, SO_2_, and SD.

Fig 2 illustrates the outcomes of local spatial autocorrelation analysis. The figure is divided into four sections, each representing a distinct type of local spatial relationship. As a result, the Moran’s *I* scatter plot primarily falls in the first and third sections, which suggests that all four variables show a positive spatial correlation. The findings suggest that regions with comparable levels of air pollution or healthcare access tend to form clusters, showing either high-high or low-low patterns. Overall, both air quality and public health across China demonstrate spatial heterogeneity and interdependence.

#### 3.1.3 Bivariate spatial autocorrelation test.

The bivariate spatial correlation analysis allows for the identification of external spatial influences related to air pollution. This method enables an assessment of how air pollutants in one province may affect public health in neighboring regions. This study further refines the analytical dimensions. It separately examines the correlations between diagnosis and treatment visits and three pollution indicators: PM_2.5_ concentration, industrial SO₂ emissions, and industrial smoke and dust. The findings are summarized in Table 5.

**Table 5:** China’s bivariate global Moran index for assessing air pollution and public health.

Year	NDT&PM_2.5_	NDT&SO_2_	NDT&SD
2000	0.263^**^	0.121^*^	0.080^**^
2001	0.243^**^	0.107^*^	0.027^**^
2002	0.207^**^	0.098^*^	0.115^**^
2003	0.285^**^	0.079^*^	0.137^**^
2004	0.259^**^	0.077^*^	0.135^**^
2005	0.233^**^	0.069^*^	0.139^*^
2006	0.270^**^	0.138^**^	0.173^**^
2007	0.262^**^	0.078^**^	0.165^**^
2008	0.233^**^	0.126^**^	0.162^*^
2009	0.244^**^	0.123^**^	0.194^**^
2010	0.284^**^	0.113^**^	0.157^*^
2011	0.294^**^	0.086^*^	0.199^**^
2012	0.231^**^	0.104^*^	0.107^**^
2013	0.294^**^	0.120^**^	0.119^**^
2014	0.242^**^	0.105^**^	0.037^**^
2015	0.248^**^	0.147^**^	0.016^*^
2016	0.227^**^	0.043^**^	0.068^**^
2017	0.262^**^	0.134^*^	0.102^**^
2018	0.226^**^	0.146^**^	0.101^**^
2019	0.205^**^	0.094^**^	0.112^**^
2020	0.263^**^	0.136^**^	0.108^**^
2021	0.235^**^	0.130^**^	0.112^**^

Note: Significance levels are indicated by

*** ,

** , and

* , representing p-values of 0.01, 0.05, and 0.1, respectively.

The findings strongly suggest a notable positive spatial relationship between PM_2.5_ levels, industrial smoke and dust, industrial SO_2_ emissions, and public health. This implies that air pollution in one province not only harms the health of its residents but also negatively affects neighboring regions, reinforcing the spatial dependence between them.

### 3.2 Empirical results

#### 3.2.1 National level analysis.

Building upon the outcomes of the previously discussed spatial econometric analysis [58], this research employs a spatial Durbin model with double fixed effects to examine how various factors influence the spatial and temporal distribution of public health [59,60]. Generally speaking, spatial Durbin models provide estimates of bidirectional fixed effects, but due to the inability of the model to directly reflect its marginal effects, assessing the direct effects of the independent variables on the dependent variable proves to be a challenging task [61]. Using the coefficients derived from the SDM regression, we calculated the direct, indirect, and total effects to evaluate how air pollution affects public health [62]. The detailed results can be found in Table 6 [63].

**Table 6:** Results of direct, indirect and total effects of air pollution on public health.

Variables	Direct effect	Indirect effect	Total effect
logPM_2.5_	0.1102^***^	0.1782^***^	0.2884^***^
	(0.0293)	(0.0640)	(0.0653)
logSO_2_	0.0375^***^	0.0189	0.0563
	(0.0102)	(0.0317)	(0.0359)
logSD	0.0043	0.1322^***^	0.1365^***^
	(0.0129)	(0.0373)	(0.0421)
logPGDP	0.5288^***^	0.2189^**^	0.7477^***^
	(0.0443)	(0.1038)	(0.1226)
CYJG	0.0063^***^	0.0119^***^	0.0182^***^
	(0.0012)	(0.0036)	(0.0044)
GLP	−0.0031^**^	−0.0153^***^	−0.0184^***^
	(0.0014)	(0.0053)	(0.0064)
logYL	−0.0697^***^	−0.1519^***^	−0.2217^***^
	(0.0165)	(0.0486)	(0.0557)
YS	−0.0125^***^	−0.0061	−0.0186^***^
	(0.0016)	(0.0051)	(0.0055)
logJY	−0.1024^***^	−0.2548^***^	−0.3573^***^
	(0.0236)	(0.0660)	(0.0683)
GZ	−0.1475^***^	−0.6346^***^	−0.7820^***^
	(0.0484)	(0.1469)	(0.1657)

Note: Statistical significance at the 0.01, 0.05, and 0.1 levels is denoted by

*** ,

** , and

* , respectively. The values in parentheses represent the robust standard errors, and “-” indicates that they are not included.

According to the decomposition results of the impact effects, from the perspective of direct effects, the significance level of PM_2.5_ concentration at 1% has been tested, indicating that a 1% rise in PM_2.5_ concentration in the local area, the number of diagnosis and treatment visits can significantly increase by 0.1102%. A rise in PM_2.5_ concentration represents an increase in diagnosis and treatment visits, leading to a decrease in public health levels. This result is both statistically and practically significant. PM_2.5_, as a type of inhalable particulate matter, is characterized by its small particle size and strong penetrability, allowing it to reach deep into the respiratory tract, infiltrate the alveoli, and even enter the bloodstream, thereby causing direct harm to human health. A substantial body of epidemiological research has demonstrated that rising PM_2.5_ concentrations significantly increase the incidence of respiratory diseases, such as asthma, chronic bronchitis, and pulmonary infections, as well as cardiovascular conditions, particularly among vulnerable groups, including the elderly, children, and individuals with preexisting chronic illnesses [64].

Consequently, a 1% increase in PM_2.5_ concentration is associated with a 0.1102% rise in the number of medical visits, indicating a high degree of sensitivity among residents to pollution-induced health risks. The statistically significant positive coefficient suggests that higher levels of air pollution exacerbate symptoms or trigger the recurrence of chronic diseases, thereby elevating the demand for healthcare services and increasing the frequency of outpatient and emergency visits [65].

The significance level of industrial SO_2_ emissions at 1% has been tested, indicating that a 1% rise in industrial SO_2_ emissions in the local area, the number of diagnosis and treatment visits can significantly increase by 0.0375%. The increase in industrial SO_2_ emissions represents an increase in diagnosis and treatment visits, leading to a decrease in public health levels. From an immediate impact standpoint, there is no notable correlation between emissions of industrial smoke and dust and the medical visits.

When considering indirect effects, a 1% rise in PM_2.5_ levels in surrounding areas is associated with a 0.1782% increase in medical consultations locally. This rise in particulate matter also leads to higher treatment visits in nearby regions, indicating a connected regional impact. A 1% rise in industrial smoke and dust emissions in nearby regions is associated with a 0.1322% increase in medical consultations locally, reflecting an indirect effect. A rise in industrial smoke and dust emissions in this region also promotes an increase in nearby diagnosis and treatment visits, which has a regional linkage effect. Considering the indirect impacts, no meaningful correlation exists between industrial SO_2_ emissions and the number of medical visits.

In the overall effect, a 1% rise in PM_2.5_ concentration and industrial smoke and dust emissions between the local area and adjacent areas, it promotes a 0.2884% and 0.1365% increase in the number of diagnosis and treatment visits in the region, which has a negative impact on public health in the region. No meaningful relationship was found between industrial SO_2_ emissions and healthcare visits. The findings align with real-world conditions. Additionally, Xie Peng [66] observed that higher concentrations of air pollutants contribute to a rise in overall mortality, particularly from respiratory diseases.

When considering the control variables, the results can be outlined as follows:

When controlling for other variables, the overall effect aligns with both the direct and indirect impacts. An increase in the share of the secondary sector within the economy negatively impacts the health of local communities. The release of industrial pollutants during secondary industry production has intensified the harmful effects of environmental contamination on public health, leading to a higher number of medical visits. As per capita GDP rises, it is often accompanied by longer working hours for the public sector, which may result in employees being exposed to poorer air quality for extended periods [67]. Consequently, this leads to a higher incidence of respiratory illnesses and an increase in hospital visits and treatments [68]. Enhancing healthcare and educational services contributes positively to public health, aiming to better protect the health level of the people. Strengthening urban greening construction plays a positive role in promoting public health. From a deeper perspective, greening can effectively purify the environment and alleviate pollution problems. Environmental protection facilities have the function of reducing particulate matter in the air, and green spaces can absorb a large amount of harmful substances in the air [15].

#### 3.2.2 Robustness test.

Robustness testing refers to the process of verifying the reliability of an evaluation method and the stability of its results in different contexts. When some initial facts and related hypotheses are changed, it is found that they are consistent with the initial results and the conclusion is considered consistent [14]. On the contrary, if there is a discrepancy between the obtained decision and the initial decision, the reasons must be identified and clarified. In this article, the adjacent geographic matrices of the original model are transformed into geographic distance matrices for robustness testing. The findings from the inspection can be found in Table 7.

**Table 7:** Robustness test results of spatial Doberman model under geographical distance matrix.

Variables	Direct effect	Indirect effect	Total effect
logPM_2.5_	0.1035^***^	0.5578^**^	0.6613^*^
	(0.0291)	(0.2387)	(0.2326)
logSO_2_	0.0349^***^	0.2443^*^	0.2792
	(0.0102)	(0.1392)	(0.0359)
logSD	0.0165	0.2514	0.2679^***^
	(0.0132)	(0.1657)	(0.0421)
logPGDP	0.4991^***^	1.1397^*^	1.6388^**^
	(0.0476)	(0.6879)	(0.7047)
CYJG	0.0067^***^	0.0153	0.0220^***^
	(0.0014)	(0.0161)	(0.0044)
GLP	−0.0035^**^	−0.0683^***^	−0.0719^***^
	(0.0015)	(0.0214)	(0.0225)
logYL	−0.0950^***^	−0.8173^**^	−0.9123^***^
	(0.0188)	(0.3195)	(0.3316)
YS	−0.0088^***^	−0.0326	−0.0414
	(0.0021)	(0.0279)	(0.0292)
logJY	−0.1200^***^	−1.4172^***^	−1.5372^***^
	(0.0225)	(0.3932)	(0.4014)
GZ	−0.1922^***^	−2.0274^***^	−2.2196^***^
	(0.0524)	(0.6736)	(0.6962)

Note: Statistical significance at the 0.01, 0.05, and 0.1 levels is denoted by

*** ,

** , and

* , respectively. The values in parentheses represent the robust standard errors, and “-” indicates that they are not included.

According to the above method, the spatial Durbin model was selected for analysis, and it was found that the sign of the model coefficients did not change, only in terms of estimated coefficients and significance. The levels of PM_2.5_, SO_2_ emissions from industries, and industrial smoke and dust all show significant positive correlations with the number of medical visits, suggesting that air pollution has contributed to a decline in public health. This means that whether using geographic distance weights or adjacency weight matrices, the signs of the main variables remain unchanged, and the coefficient sizes slightly change, but there is no substantial change. This suggests that the results of the empirical analysis in this paper demonstrate strong reliability.

#### 3.2.3 Regional differences.

As previously mentioned, elements like air quality, economic development, and social conditions all play a significant role in shaping public health outcomes. Due to its vast size, China experiences considerable disparities in urbanization, industrial development, infrastructure, regional economic prosperity, and climate across its eastern, central, and western regions [69]. This study examines the geographic effects of air pollution on public health by categorizing the areas into eastern, central, and western regions [69], then analyzing the findings. These regional classifications align with the segmentation used by China’s National Bureau of Statistics [70,71].

Table 8 presents the regression outcomes across the three regions. The impact of air pollution on the health of local populations differs considerably across these areas. When examining the factors that influence the medical visits in the eastern, western, and central regions, it was observed that the effect of PM_2.5_ levels on public health is most pronounced in the eastern region, showing marked significance. Specifically, when there is a 1% change in PM_2.5_ concentration, the change in the number of diagnosis and treatment visits, which is an important indicator of public health, in the eastern region is 0.2516%. Industrial SO_2_ emissions have a notable effect on public health, particularly in the eastern and central areas. A 1% increase in these emissions leads to a 0.0819% rise in medical consultations in the eastern region, and a 0.1223% increase in the central region. Similarly, the release of industrial smoke and dust significantly influences public health in both the western and central regions. A 1% variation in industrial smoke and dust emissions leads to a change of 0.1256% in the number of medical visits in the central region, and 0.0469% in the western region, which serves as a key indicator of public health.

**Table 8:** The Direct, Indirect, and Total Effects of Air Pollution on Public Health in the Eastern, Central, and Western Regions.

Variables	Eastern region			Central region			Western region		
Direct effect	Indirect effect	Total effect	Direct effect	Indirect effect	Total effect	Direct effect	Indirect effect	Total effect
logPM_2.5_	0.0596	0.1920^*^	0.2516^*^	0.0424	0.0288	0.0712	0.0695^**^	0.0310	0.1005
	(0.0652)	(0.1039)	(0.1223)	(0.0604)	(0.0698)	(0.0473)	(0.0350)	(0.0684)	(0.0740)
logSO_2_	0.0404^**^	0.0414	0.0819^*^	0.0031	0.1192^***^	0.1223^***^	0.0516^***^	0.0565^*^	0.1081
	(0.0172)	(0.0320)	(0.0425)	(0.0243)	(0.0389)	(0.0378)	(0.0137)	(0.0290)	(0.0296)
logSD	0.0474^**^	−0.0639	−0.0165	0.0799^**^	0.0457	0.1256^*^	0.0350^**^	0.0119	0.0469^*^
	(0.0228)	(0.0431)	(0.0576)	(0.0350)	(0.0443)	(0.0535)	(0.0161)	(0.0263)	(0.0248)
logPGDP	0.1955^**^	0.1246	0.3201^*^	0.3781^***^	0.3419^**^	0.7200	0.6967^***^	0.0984	0.7951^***^
	(0.0803)	(0.1328)	(0.1771)	(0.1234)	(0.1553)	(0.1672)	(0.0890)	(0.1562)	(0.1929)
CYJG	0.0095^***^	0.0173^**^	0.0267^***^	0.0021	0.0004	0.0025	0.0059^***^	0.0089^***^	0.0148^***^
	(0.0035)	(0.0070)	(0.0094)	(0.0024)	(0.0023)	(0.0035)	(0.0018)	(0.0034)	(0.0042)
GLP	−0.0062^*^	−0.0114	−0.0176	−0.0067^***^	0.0003	−0.0064	−0.0055^***^	−0.0020	−0.0075^**^
	(0.0037)	(0.0080)	(0.0109)	(0.0024)	(0.0052)	(0.0062)	(0.0013)	(0.0030)	(0.0036)
logYL	−0.1667^***^	−0.2369^***^	−0.4036^***^	−0.1117^**^	−0.4797^***^	−0.5913^***^	−0.0182	−0.1746^***^	−0.1929^***^
	(0.0365)	(0.0770)	(0.1000)	(0.0527)	(0.0719)	(0.1050)	(0.0203)	(0.0483)	(0.0532)
YS	−0.0125^***^	−0.0043	−0.0168^*^	−0.0207^***^	−0.0016	−0.0223^***^	−0.0078	−0.0098	−0.0176
	(0.0039)	(0.0063)	(0.0091)	(0.0037)	(0.0045)	(0.0050)	(0.0050)	(0.0101)	(0.0115)
logJY	−0.2139^***^	−0.2131^*^	−0.4270^***^	−0.1270^*^	−0.1893^**^	−0.3163	−0.0175	−0.0695	−0.0870
	(0.0573)	(0.1167)	(0.1358)	(0.0650)	(0.0926)	(0.1260)	(0.0353)	(0.0807)	(0.0877)
GZ	−0.0600	−0.0337	−0.0937	−0.2333^***^	−0.1128	−0.3461^**^	−0.0823	−0.1952	−0.2775
	(0.1174)	(0.1949)	(0.2711)	(0.0691)	(0.1304)	(0.1385)	(0.0703)	(0.1218)	(0.1152)

Note: Statistical significance at the 0.01, 0.05, and 0.1 levels is denoted by

*** ,

** , and

* , respectively. The values in parentheses represent the robust standard errors, and “-” indicates that they are not included.

Overall, the eastern region experiences less adverse health effects from air pollution compared to central China. This can be explained by the faster economic growth in the east and the adoption of more advanced technologies for environmental protection, which lead to more effective control of exhaust emissions. Additionally, certain provinces along the eastern coast possess stronger natural purification capabilities that assist in more efficiently eliminating airborne pollutants, thereby diminishing their harmful effects on public health. The combined influence of these factors results in a relatively improved public health situation in the eastern region, which is better equipped to handle the challenges of air pollution. Pollution is more detrimental in the central area, largely as a result of the extensive coal mining activities that dominate the region. The extensive burning of coal in this area has led to significant air quality issues. Moreover, the central part of China is home to a high concentration of industrial activity, which contributes to significant emissions of industrial waste gases. This, consequently, this exacerbates the adverse health impacts of pollution on the local population. The air quality challenges in the central region are exacerbated by a range of underlying causes, leading to detrimental effects on the health of local residents [72]. Currently, the western region experiences less air pollution-related health impact than the central and eastern areas. This is primarily due to the region’s industrial sector is still in the early stages of growth, resulting in relatively less industrial waste gas emissions. In addition, the western region has a vast area and sparse population distribution, with relatively low urban population density. The reason for the relatively mild consequences of air pollution on local public health in this area can be largely attributed to this factor.

#### 3.2.4 Policy implications.

Building on the findings presented earlier, policymakers in China and other developing countries can draw upon several recommendations for action:

(1) In order to encourage cross-regional healthy development in a targeted way, public health policies must be flexibly formulated based on local features, given the variations in actual situations in different regions. Given the significant regional disparities in the health impacts of air pollution across eastern, central, and western China, region-specific policy approaches are essential. The eastern region should consolidate existing achievements, promote green and low-carbon transformation, and enhance both inter-regional coordination and refined pollution monitoring to prevent rebound effects. In the central region, efforts should focus on reducing coal consumption, optimizing industrial structures, and establishing health risk early-warning systems to strengthen public health preparedness. The western region, where pollution levels remain relatively low, should seize this window of opportunity to promote green industrial development, and improve basic healthcare systems to enhance environmental resilience. A differentiated regional governance strategy will facilitate a synergistic outcome between pollution control and public health protection.
(2) To reduce the harmful impacts of air pollution, stronger measures should be implemented at the source, with an ongoing effort to improve pollution control strategies. In addition, differentiated regional air pollution control policies can be formulated based on local conditions to develop regional plans for preventing and controlling air pollution [73], strictly controlling the emission of air pollutants within the region, and implementing subsidies and tax policies that match environmental pollution reduction to ensure the effective implementation of the plan.
(3) Given how crucial income is to enhancing public health, we should give income distribution concerns a lot of thought and concentrate on making income distribution reforms as effective as possible. Simultaneously, attempts should be made to raise the share of household income in the overall national income in order to more fairly benefit everyone from development accomplishments. Residents’ capacity to prevent illnesses is positively impacted by an increase in their economic level. Firstly, the increase in residents’ income can alleviate the pressure of medical expenses, thereby alleviating the problem of difficulty in seeking medical treatment. Secondly, increasing residents’ income can help increase investment in disease prevention, such as purchasing air purification devices, raising awareness of disease prevention, and reducing the incidence of diseases.
(4) We should focus more on the education sector and follow the strategic policy of reviving the nation via research and education, given the role that education plays in advancing public health. All levels of education should receive more funding, and social capital should be judiciously directed to contribute to educational advancement. Actively promote educational institutions to carry out environmental health education activities, increase the public’s understanding of air pollution’s impact and the long-term health risks through measures such as information dissemination and behavioral intervention, cultivate health awareness, reduce behaviors that are not conducive to air pollution prevention, and enhance awareness and behavior of air pollution prevention.

### 3.3 Strengths and limitations of the study

The issue of effectively managing air pollution while enhancing public health has sparked considerable discussion in both academic circles and policy-making bodies. This study investigates the relationship between public health and air pollution across 31 Chinese provinces from 2000 to 2021. By constructing a spatial Durbin panel model, it explores how hospital visits for diagnosis and treatment respond to three key pollutants—PM_2.5_ concentration, industrial SO₂ emissions, and industrial smoke and dust—while controlling for socioeconomic and healthcare-related factors. The analysis further distinguishes regional differences across eastern, central, and western China.

The innovations of this paper are threefold. First, it adopts a multi-pollutant perspective rather than focusing on a single pollutant, offering a more holistic view of how different sources of air pollution jointly affect health outcomes. Second, it introduces a spatial econometric framework, capturing not only local effects but also spatial spillovers from neighboring regions, which better reflects the transboundary nature of air pollution. Third, this study emphasizes the regional heterogeneity of the impact of air pollution on health by comparing the eastern, central, and western regions. These innovations allow the paper to go beyond correlation analysis and offer spatially informed, policy-relevant insights for region-specific public health and environmental governance strategies.

While this study provides valuable insights into the relationship between air pollution and public health, several limitations should be acknowledged. First, the use of hospital visits as a proxy for health outcomes presents certain constraints, as it does not allow for the identification of specific diseases directly attributable to air pollution exposure. Future research could incorporate more disaggregated health data—such as disease-specific or cause-specific morbidity statistics—to more accurately assess the impacts of air pollution on cardiovascular and respiratory health. Second, the health records used in this study do not include information on patients’ residential locations. As a result, it is not possible to determine the origin of each patient, and some individuals may seek care in regions with more developed medical infrastructure, especially in large cities, regardless of where their pollution exposure occurred. This may lead to a spatial mismatch between environmental exposure and health outcomes. Third, the data may capture cases triggered by indoor air pollution, which is beyond the scope of this study and may introduce potential confounding in estimating the effects of ambient air pollution.

## 4. Conclusions

The following points highlight the major conclusions drawn from the research:

First, the Moran’s index for both hospital visits and air pollution is found to be above 0, and it generally shows statistical significance at the 10% level. This indicates a direct connection between the number of hospital visits and air pollution levels across different provinces. Simultaneously, the spatial patterns primarily emerge as clusters of similar values, characterized by high values grouped with high values and low values with low values. The bivariate correlation analysis reveals that the global Moran’s index exceeds 0, and the annual significance values under each scenario are basically significant, showing that industrial SO_2_ pollution and NDT, PM_2.5_ pollution and NDT, and industrial smoke and dust pollution and NDT have a spatially positive connection. Next, the findings from the spatial econometric models at the provincial level indicate that rising levels of air pollutants significantly contribute to the increase in medical visits. This further supports the idea that air pollution significantly contributes to the decline in public health. Third, considering other socio-economic factors, this study included other control variables. The positive and significant effect of per capita GDP on healthcare visits suggests that as China’s economy grows, there is a rising awareness among residents about their health, resulting in more frequent hospital visits. As economic development progresses, the expansion of the secondary industry inevitably leads to the production of air pollutants, which can negatively affect public health. In contrast, the extent of green space in urban areas serves as a key environmental factor, contributing positively to the well-being of the population. An increase in local government spending on healthcare and a higher number of practicing doctors have contributed to expanding access to medical services across China, thereby boosting the number of hospital visits. Enhancing medical security is designed to more effectively protect public health. Likewise, increased spending on education plays a crucial role in significantly boosting public health outcomes. Finally, the impact of air pollution on human health varies depending on the region, with distinct differences observed in the eastern, western, and central parts of China. In particular, the western region experiences a relatively lesser impact from air pollution compared to the central and eastern regions [74].

## Supporting information

S1 Fig The graphical abstract of this article, which visually displays the key results and methods of the research. (TIF)
