# Supplementary figures and images for: The spatial and dynamic impact of air pollution on public health: Evidence from China 2000–2021
Source: PLoS One. 2025 Jul 7;20(7):e0327319. doi: 10.1371/journal.pone.0327319 (PMC12233298; doi:10.1371/journal.pone.0327319)

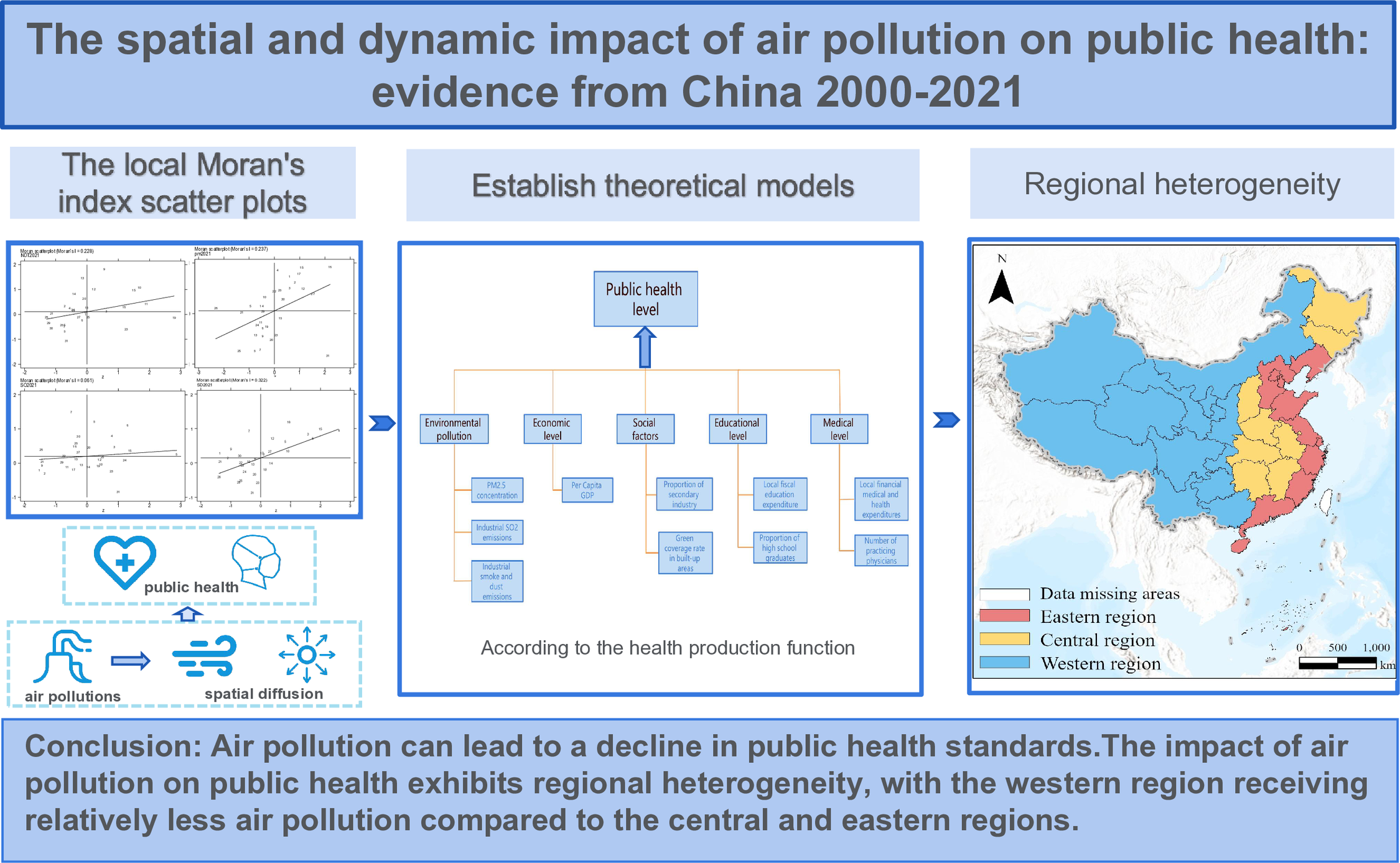

Supplement: S1 Fig — (TIF) [file pone.0327319.s001.tif]
